# Identification and characterization of MUS81 point mutations that abolish interaction with the SLX4 scaffold protein

**DOI:** 10.1016/j.dnarep.2014.08.004

**Published:** 2014-12

**Authors:** Nidhi Nair, Dennis Castor, Thomas Macartney, John Rouse

**Affiliations:** MRC Protein Phosphorylation and Ubiquitylation Unit, College of Life Sciences, University of Dundee, Dundee DD1 5EH, Scotland, UK

**Keywords:** SLX4, MUS81, Nuclease, Scaffold, Inter-strand crosslink

## Abstract

MUS81-EME1 is a conserved structure-selective endonuclease with a preference for branched DNA substrates *in vitro* that correspond to intermediates of DNA repair. Cells lacking MUS81 or EME1 show defects in the repair of DNA interstrand crosslinks (ICL) resulting in hypersensitivity to agents such as mitomycin C. In metazoans, a proportion of cellular MUS81-EME1 binds the SLX4 scaffold protein, which is itself instrumental for ICL repair. It was previously reported that mutations in SLX4 that abolished interaction with MUS81 affected ICL repair in human cells but not in murine cells. In this study we looked the other way around by pinpointing amino acid residues in MUS81 that when mutated abolish the interaction with SLX4. These mutations fully rescued the mitomycin C hypersensitivity of MUS81 knockout murine cells, but they were unable to rescue the sensitivity of two different human cell lines defective in MUS81. These data support an SLX4-dependent role for MUS81 in the repair, but not the induction of ICL-induced double-strand breaks. This study sheds light on the extent to which MUS81 function in ICL repair requires interaction with SLX4.

## 1 Introduction

MUS81-EME1 is an evolutionarily conserved structure-selective endonuclease with a preference *in vitro* for branched DNA substrates such as 3′ flaps, replication forks and nicked Holliday junctions (HJs) that correspond to intermediates of DNA repair [1]. Cells lacking MUS81 or EME1 show defects in the repair of DNA interstrand crosslinks (ICL) resulting in hypersensitivity to agents such as mitomycin C and platinum compounds [2], [3], [4], [5]. In metazoans, a proportion of the total cellular pool of MUS81-EME1 binds the SLX4 scaffold protein [6]. This scaffold is the central coordinator of a DNA repair toolkit that includes two other endonucleases: XPF-ERCC1 and SLX1 [7], [8], [9], which are also required for ICL repair, together with SLX4 [7], [8], [9], [10], [11], [12].

Although both SLX4 and MUS81-EME1 are highly conserved, budding yeast Slx4 does not associate with Mus81-Mms4^Eme1^ [13]. So somewhere in evolution SLX4 and MUS81 developed an ability to interact with each other. Furthermore the interaction of SLX4 with MUS81-EME1 appears to be restricted to late S and G2 phases of the cell cycle [14]. Also, mutations in murine SLX4 that disrupt the interaction with MUS81 largely abolishes the resolution of Holliday junctions that escape dissolution but does not affect ICL repair [11], [12]. In this study, we mapped domains and residues in MUS81 required for binding to SLX4 and tested the consequences for ICL repair.

## 2 Results and discussion

### 2.1 Mapping the SLX4 interacting region in MUS81

**Fig. 1:**
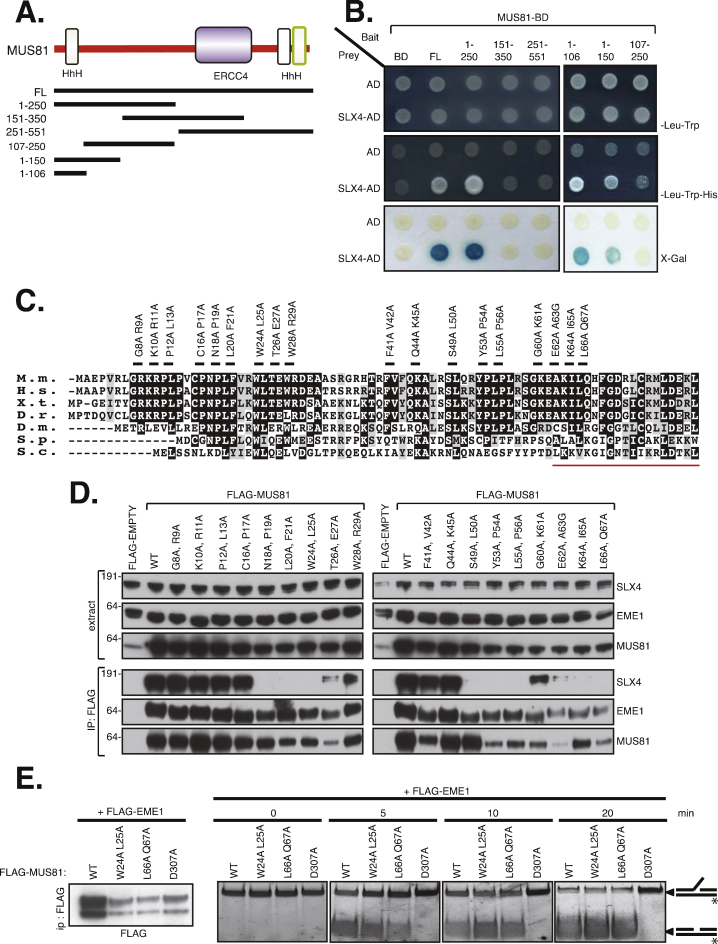
Identification of MUS81 mutants that abolish interaction with SLX4. (A) MUS81 fragments used in the yeast two-hybrid assay. (B) Yeast were co-transformed with the bait and prey plasmid combinations indicated and plated on the media lacking leucine and tryptophan, with or without histidine (top and middle panels) or onto X-Gal (bottom panel). Y2H assays were performed with a GAL4 DNA binding domain (BD) fusion of MUS81 and activation domain (AD) fusion of SLX4 to detect interaction between these proteins. Cells grown on medium lacking LEU and TRP (to select for bait and prey plasmids) were replica-plated to medium lacking LEU, TRP and HIS to test for activation of the *HIS3* reporter gene or tested for *lacZ* reporter gene activity. (C) Alignment of the N-terminus of MUS81 from different species. Residues were mutated in pairs of two to alanine (black lines). The residues that constitute the Helix-hairpin-Helix (HhH) are underlined in red. M.m., *Mus musculus*; H.s., *Homo sapiens*; X.t., *Xenopus tropicalis*; D.r., *Danio rerio*; D.m., *Drosophila melanogaster*; S.p., *Saccharomyces pombe*; S.c., *Saccharomyces cerevisiae*. (D) HEK293 cells were transiently transfected with plasmids expressing FLAG-EMPTY, FLAG-MUS81 wild-type (WT) or FLAG-MUS81 carrying double point mutants as shown in (C). Extracts were subjected to western blotting to test expression (upper panel) or immunoprecipitation with anti-FLAG antibodies (lower panel). (E) Immunoprecipitates from HEK293 cells co-transfected with plasmids expressing FLAG-MUS81 (WT), nuclease dead mutant FLAG-MUS81 D307A (D307A), FLAG-MUS81 loss-of-interaction mutants W24A L25A or L66A Q67A and FLAG-EME1 (EME1) were subjected to a nuclease assay with FITC-labeled 3′ flap substrate for time indicated, and the reaction products resolved by gel electrophoresis. FITC-labeled 3′ flap substrate and the cleaved duplex are shown on the right. Western blot confirming the immunoprecipitation with FLAG M2 beads is shown in the left panel.

It was previously established that it is the N-terminus (amino acids 1–86) of the MUS81 subunit of MUS81-EME1 that interacts with SLX4 [7]. To narrow down the domain in human MUS81 interacting with SLX4, a series of MUS81 fragments were cloned into a yeast two-hybrid (Y2H)-compatible vector (Fig. S1), fused to a GAL4-DNA binding domain (MUS81-BD): amino acids (1–250), (151–350) and (251–551), as well as full-length MUS81 (FL) (Fig. 1A). A Y2H screen for interaction with full-length SLX4 fused to a GAL4-activation domain (SLX4-AD) revealed that the MUS81 fragment (1–250) was capable of interaction with SLX4 (Fig. 1B). To further narrow down the SLX4-binding region of MUS81, we subdivided this region and found that a MUS81 fragment coding for amino acids 1–106 is sufficient for interaction with SLX4 (Fig. 1A and B).

### 2.2 Reverse yeast two-hybrid assay

Low fidelity mutagenic PCR was used to generate mutations in MUS81 (1–106) fragment, which was recombined into the Y2H-compatible vector as a fusion with a kanamycin resistance gene. Resulting MUS81 (1–106) mutants with stop codons or frameshift mutations in the MUS81 coding sequence were eliminated by selection at an optimized kanamycin concentration (Fig. S2). The reporter strain Mav203 has, in addition to *HIS3* and *lacZ*, a *URA3* reporter gene driven by *GAL4* sites. *URA3* encodes orotidine 5-phosphate decarboxylase (ODCase) that converts 5-fluoroorotic acid (5-FOA) to 5-fluorouracil causing cell death when plated on medium containing 5-FOA and thereby selecting for loss-of-interaction between bait/prey proteins. To this end, Mav203 yeast were co-transformed with the MUS81 (1–106)-BD allele library and SLX4-AD and plated on medium containing 5-FOA. Of the hundreds of colonies that grew on 5-FOA, around 30 were picked, re-streaked and taken forward for further analysis. A representative group of ten such clones is shown in Fig. S3A. Sequencing revealed two single point mutations at residue Leu25 (L25) and Leu47 (L47), which were chosen for further analysis based on a high frequency of occurrence. To confirm loss-of-interaction with SLX4, HEK293 cells were transiently transfected with full-length FLAG-MUS81 carrying mutations at residues Leu25 and Leu47 (L25P, L25K, L47P, L47K and L25A, L47A). Immunoprecipitation using FLAG-M2 agarose beads revealed loss-of-interaction with SLX4 compared with wild-type MUS81 (Fig. S3B).

### 2.3 Alanine scanning of conserved residues in MUS81 (1–106) fragment

As a complementary approach, we carried out alanine scanning across conserved residues in the N-terminal (1–106) HhH-bearing region conserved in a range of MUS81 orthologues [15], [16]. Pairs of conserved residues were mutated to alanine, one pair at a time (Fig. 1C) in full-length FLAG-tagged MUS81, and HEK293 cells were transiently transfected with these mutants or wild-type FLAG-MUS81. As shown in Fig. 1D, several MUS81 mutants were identified in this way that could not interact with endogenous SLX4. Two of the mutants identified *i.e.* W24A L25A and L66A Q67A, did not seem to be defective in EME1 interaction when levels of MUS81 were taken into account and were chosen for further analysis. Leu25 was also identified in the reverse yeast two-hybrid assay (see above).

### 2.4 Effect of loss-of-SLX4-interaction mutants on MUS81 nuclease activity

Most of the cellular complement of MUS81-EME1 does not interact with SLX4, and we thought it unlikely that preventing interaction with SLX4 would affect the nuclease activity of MUS81-EME1. To test this, HEK293 cells were co-transfected with FLAG-MUS81 wild-type (WT) or MUS81 mutants (W24A L25A and L66A Q67A) that could not interact with SLX4 - and FLAG-EME1. As control a nuclease dead mutant FLAG-MUS81 D307A (D307A) was used. Anti-FLAG immunoprecipitates from cells expressing wild-type MUS81 cleaved the 3′ flap substrate efficiently, but not from cells expressing the nuclease dead D307A mutant (Fig. S4). As shown in Fig. 1E, 3′ flap nuclease activity in FLAG immunoprecipitates in the context of the MUS81 mutants W24A L25A and L66A Q67A that did not interact with SLX4 was indistinguishable from wild-type MUS81.

### 2.5 Impact of loss-of-SLX4-interaction MUS81 mutants on ICL repair in mouse cells

**Fig. 2:**
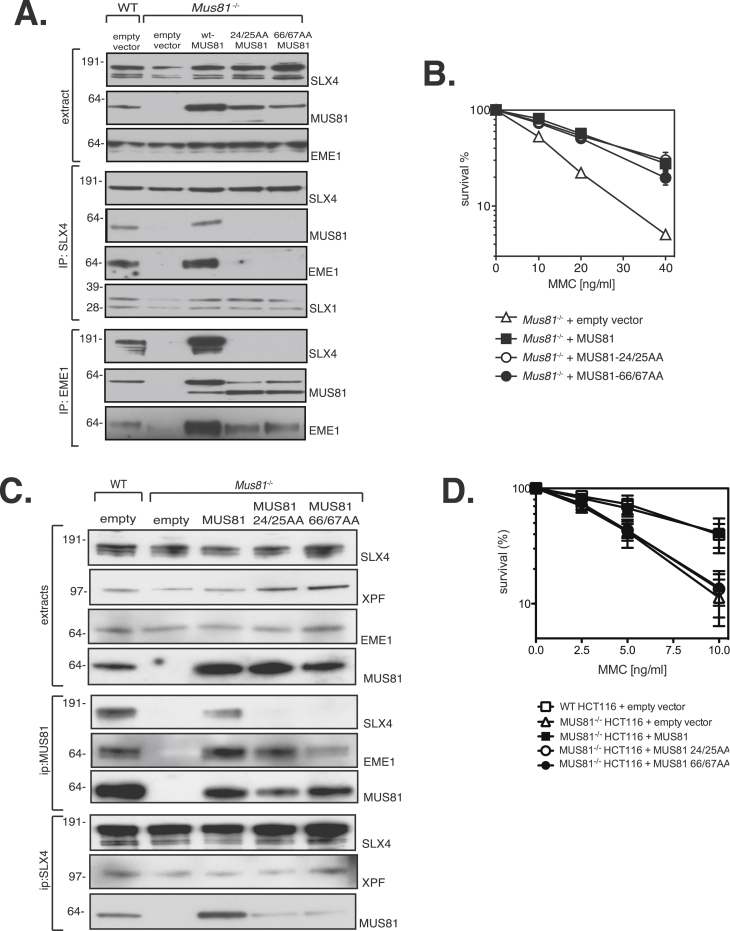
MUS81 mutations that cannot interact with SLX4 cause ICL repair defects in human cells but not mouse cells. (A) *Mus81*^−/−^ MEFs were infected with retroviruses expressing wild-type MUS81, MUS81 24/25AA or MUS81 66/67AA. Wild-type MEFs (WT) and *Mus81*^−/−^ MEFs infected with empty virus were used as controls. Extracts were subjected to western blotting to test expression (upper panel) or immunoprecipitation with anti-SLX4 (middle panel) and anti-EME1 antibodies (lower panel). (B) Clonogenic survival analysis of *Mus81*^−/−^ MEFs stably expressing MUS81, MUS81 24/25AA or MUS81 66/67AA, exposed to MMC. For each genotype, cell viability of untreated cells was defined as 100%. *Mus81*^−/−^ MEFs infected with empty virus were used as controls. Data are represented as mean ± SEM, *n* = 3. (C) *MUS81*^−/−^ HCT116 cells were infected with retroviruses expressing MUS81, MUS81 24/25AA or MUS81 66/67AA. Wild-type cells (WT) and *MUS81*^−/−^ HCT116 cells infected with empty virus were used as controls. Extracts were subjected to western blotting to test expression (upper panel) or immunoprecipitation with anti-MUS81 and anti-SLX4 antibodies (lower panel). (D) Clonogenic survival analysis of *MUS81*^−/−^ HCT116 cells stably expressing MUS81, MUS81 24/25AA or MUS81 66/67AA, exposed to MMC. For each genotype, cell viability of untreated cells was defined as 100%. Wild-type HCT116 and *MUS81*^−/−^ HCT116 cells infected with empty virus were used as controls. Data are represented as mean ± SEM, *n* = 3.

We next checked the ability of MUS81 mutants to rescue MMC hypersensitivity of *Mus81*^−/−^ MEFs. To this end *Mus81*^−/−^ MEFs [5] were infected with retroviruses for stable expression of wild-type mouse MUS81 (wt-MUS81) or full-length mouse MUS81 carrying the mutations W24A L25A (MUS81 24/25AA) or L66A Q67A (MUS81 66/67AA). Wild-type and *Mus81*^−/−^ MEFs were infected with empty viruses to serve as controls. MUS81 expression in the complemented *Mus81*^−/−^ MEFs was assessed by immunoblotting of whole cell extracts (Fig. 2A, top panel). While wild-type MUS81 and EME1 were detected in SLX4 immunoprecipitates, the MUS81 loss-of-SLX4-interaction mutants *i.e.* MUS81 24/25AA and MUS81 66/67AA were not (Fig. 2A, middle panel). Similar results were obtained when endogenous EME1 was precipitated from cells complemented with the MUS81 mutants (Fig. 2A, bottom panel). SLX4 was detected in EME1 immunoprecipitates from cells expressing wild-type MUS81, but not MUS81 24/25AA and MUS81 66/67AA mutants. The complemented *Mus81*^−/−^ MEFs were used in a clonogenic survival assay to test sensitivity to MMC. *Mus81*^−/−^ MEFs are hypersensitive to MMC compared to wild-type MEFs. This sensitivity was rescued by ectopic expression of wild-type MUS81, and the MUS81 24/25AA and MUS81 66/67AA mutants were indistinguishable from wild-type (Fig. 2B). These data suggest that the role of MUS81 in ICL repair is independent of its interaction with SLX4 at least in murine cells.

### 2.6 MUS81 mutants that cannot interact with SLX4 show defective ICL repair in human HCT116 cells

We tested the ability of the MUS81 24/25AA and MUS81 66/67AA mutants to rescue the ICL hypersensitivity of human HCT116 colon cancer cells in which the MUS81 gene had been disrupted [4]. To this end wild-type MUS81 or MUS81 24/25AA and 66/67AA mutants were expressed in *MUS81*^−/−^ HCT116 cells. Wild-type and *MUS81*^−/−^ HCT116 cells transduced with empty vectors were used as controls. As in MEFs, the MUS81 loss-of-interaction mutants failed to interact with SLX4 in co-immunoprecipitation experiments (Fig. 2C). Surprisingly, the 24/25AA and 66/67AA mutants were incapable of rescuing MMC sensitivity in *MUS81*^−/−^ HCT116 cells (Fig. 2D) in contrast to *Mus81*^−/−^ MEFs where MUS81 interaction with SLX4 was dispensable for ICL repair.

### 2.7 MUS81 mutants that cannot interact with SLX4 show defective ICL repair in HEK293 cells

**Fig. 3:**
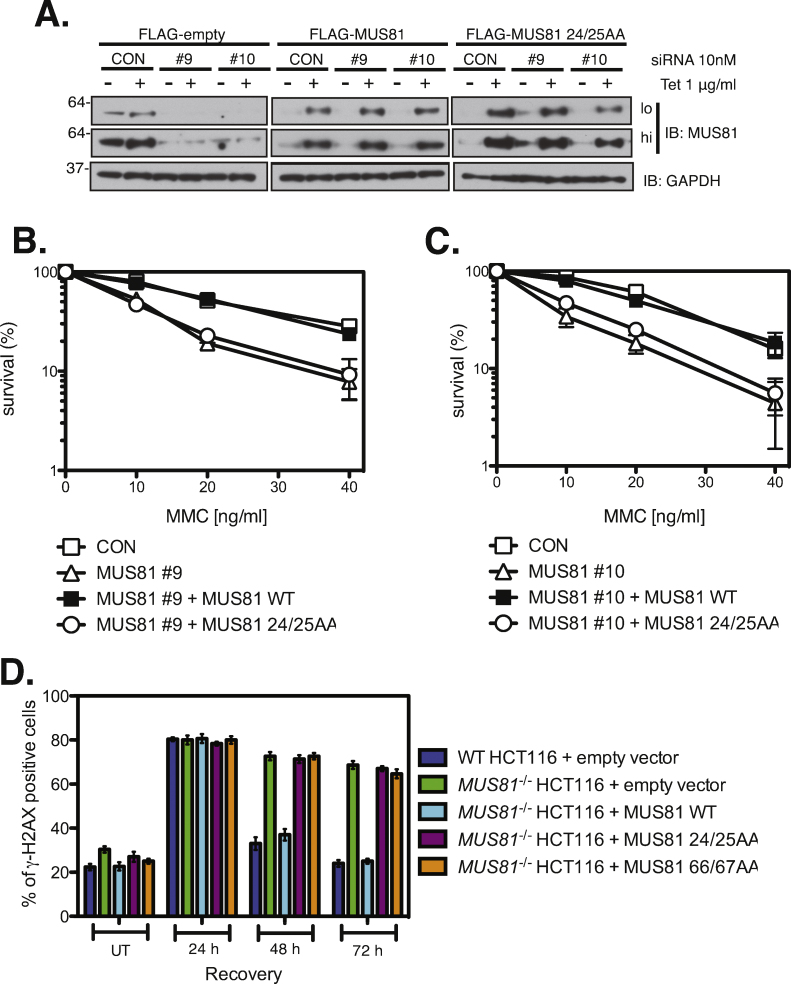
Interaction of MUS81-EME1 with SLX4 is required for repair of ICL-induced DSBs in human cells. (A) HEK293 cells were transfected for stable expression of FLAG-empty or siRNA protected FLAG-MUS81 or FLAG-MUS81 24/25AA under a tetracycline inducible promoter. These cells were then transfected with either scrambled (CON) or MUS81 specific siRNA oligos (#9, #10), following which expression of FLAG-tagged MUS81 (wild-type or mutant) was induced. MUS81 expression was blotted using anti-MUS81 antibodies. GAPDH expression served as control. (B) HEK293 cells stably expressing FLAG-only vector were transfected with either scrambled (CON) or MUS81 specific siRNA (MUS81 #9). Alternatively, HEK293 cells stably expressing wild type FLAG-MUS81 (MUS81 WT) or FLAG-MUS81 24/25AA (MUS81 24/25AA) were knocked down for endogenous MUS81 expression using MUS81 specific siRNA (MUS81 #9). Clonogenic survival analysis of said HEK293 cells was performed following exposure to MMC. For each sample, cell viability of untreated cells was defined as 100%. Data are represented as mean ± SEM, *n* = 3. (C) Same as in (B) except cells were treated with MUS81 specific siRNA #10 (MUS81 #10). For each sample, cell viability of untreated cells was defined as 100%. Data are represented as mean ± SEM, *n* = 3. (D) Wild-type, *MUS81^−/−^* or MUS81 (wild-type or mutant) complemented *MUS81^−/−^* HCT116 cells were treated with MMC (20 ng/ml) for 16 h and then allowed to recover for the times indicated. The proportion of cells in each population with more than five γH2AX foci at each time point (γH2AX positive) was determined by immunofluorescence.

To exclude the possibility that the discrepancies observed were unique to a particular cell type, we wished to test at least one more human cell line. To this end, two separate MUS81 specific siRNAs (#9, #10) were used to deplete MUS81 from HEK293 cells and a scrambled siRNA (CON) was used as control. HEK293 cells were also transfected with plasmids that allowed stable expression of FLAG–MUS81 or FLAG–MUS81 24/25AA, under the control of a tetracycline–inducible promoter. These plasmids encoded MUS81 with conservative mutations that rendered it refractory to siRNAs #9 and #10. Endogenous MUS81 was knocked down using siRNA oligos and expression of FLAG-MUS81 (wild-type or mutant) was induced by tetracycline. HEK293 cells with a stable expression of a FLAG-empty tag, also under control of a tetracycline-inducible promoter, were used as a negative control. The knockdown of endogenous MUS81 and simultaneous expression of FLAG-tagged MUS81 (wild type or mutant) was confirmed using western blotting (Fig. 3A). These cells were then subjected to clonogenic survival assays. Depletion of MUS81 using either siRNA #9 or #10 caused HEK293 cells to be hypersensitive to MMC, which could be rescued by wild-type MUS81 in each case, but not by MUS81 24/25AA mutant (Figs. 3B and C).

### 2.8 SLX4 interaction with MUS81 is required for repair of DSBs induced by MMC in human cells

MUS81 has been implicated in generation of ICL-induced DSBs [17]. However, as shown in Fig. 3D, we found that MUS81 is not required for DSB induction in HCT116 cells, which is consistent with a previous study in *Mus81*^−/−^ MEFs that showed that ICL-induced DSBs could occur even in the absence of MUS81 [3]. The same study also showed that γH2AX foci, a widely used marker for DSBs, persisted for longer in MEFs lacking MUS81 [3]. Thus, we next wanted to test if *MUS81*^−/−^ HCT116 cells complemented with MUS81 loss-of-interaction mutants were similarly defective in repair of ICL-induced DSBs.

To this end, the disappearance of γH2AX foci following a pulse of MMC treatment was analyzed in wild-type HCT116, *MUS81*^−/−^ HCT116 or *MUS81*^−/−^ HCT116 cells complemented with MUS81 (wild-type or mutant). We observed an elevated number of *MUS81*^−/−^ HCT116 cells that were positive for γH2AX foci prior to MMC treatment as compared to wild-type HCT116 cells, which was consistent with a previous study of *Mus81*^−/−^ MEFs [3]. Following MMC treatment, the proportion of cells positive for γH2AX foci peaked at ∼80% at 24 h in all cell lines tested. Subsequently, the number of both wild-type HCT116 cells and *MUS81*^−/−^ HCT116 cells + MUS81 declined to basal levels within 48 h of drug treatment. However, for *MUS81*^−/−^ HCT116 cells and *MUS81*^−/−^ HCT116 cells complemented with MUS81 mutants that could not interact with SLX4, the percentage of cells with γH2AX foci remained constant at ∼70% for the duration of the experiment. This observation suggests that in human cells, MUS81-EME1 is required for ICL-induced DSB repair and this role of MUS81-EME1 is dependent on its interaction with SLX4.

## 3 Discussion

At present the basis for the clear difference between human and murine cells is unclear, but this is not the first evidence of a disparity between MUS81 function in mice and humans. For example, studies in *MUS81*^−/−^ HCT116 cells show that response to camptothecin (CPT) is dependent on MUS81 whereas, a separate study showed that *Mus81*^−/−^ MEFs were resistant to CPT treatment [3], [18]. Further evidence in favor of a functional disparity in ICL repair comes from studies in mutant mice models for FANC genes. While, cells isolated from these mice are ICL-sensitive, most mice do not present developmental defects, anemia and cancer predisposition that are commonly described in FANC patients [19]. Hence, while mouse models are important tools to study the mechanistic background of FA, it is important to keep in mind that there are differences in DNA repair between mice and humans.

MUS81 has been implicated in ICL-induced DSB formation as well as during homologous recombination in the latter stages of crosslink repair [3], [17]. Here we found that DSBs induced by MMC treatment were not dependent on MUS81, an observation made previously for SLX4 [6], [8]. However, MUS81 mutations that do not interact with SLX4 cause delayed repair of the DSBs as judged by γH2AX foci. This is consistent with a role for SLX4-bound MUS81-EME1 in processing repair intermediates at late stages of ICL repair, perhaps during homologous recombination.

## 4 Experimental procedures

### 4.1 Nuclease assays

Oligonucletotides were purchased from VBC Biotech. The sequences of the oligonucleotides used to prepare the 3′ flap DNA substrate are given below:

Oligo 1: 5′-AGCTACCATGCCTGCACGAATTAAGCAATTCGTAATCATGGTCATAGCT
Oligo 4: 5′-AATTCGTGCAGGCATGGTAGCT
Oligo 6: 5′-AGCTATGACCATGATTACGAATTGCTTGGAATCCTGACGAACTGTAG

Oligo 1 was labeled with fluorescein isothiocyanate (FITC) at its 5′ end. The 3′ flap substrate was prepared as previously described in [20]. After immunoprecipitation, FLAG M2 beads were washed in cell lysis buffer followed by a final wash in nuclease buffer (20 mM Tris-HCl [pH 7.5], 50 mM NaCl, 10 mM MgCl_2_, 0.1 mg/ml BSA) and assay was performed as described in [8]. Reaction products were analyzed by native PAGE (10% polyacrylamide) in TAE buffer. Gels were exposed to FLA5000IR, 473 nm, 600 V and viewed using AIDA image software.

### 4.2 Clonogenic survival analysis

MEFs or HCT116 cells were seeded in triplicates in 10 cm dishes and were allowed to attach before treatment. MMC was added to cells for 24 h before medium was replaced with fresh growth medium. HEK293 cells were seeded in 10 cm dishes at 25% confluence and allowed to adhere overnight. Cells were transfected with the relevant siRNA for 48 h and cells were split and seeded in 10 cm dishes (3500 cells/dish). Cells were allowed to adhere for a minimum of 8 h before MMC treatment for 24 h. After 10 days cells were washed, fixed and stained with Giemsa. The number of colonies with >50 cells were counted. Results were normalized to plating efficiency.

## Conflict of interest

The authors have no conflict of interest.
