# Supplementary material for: Identification and characterization of MUS81 point mutations that abolish interaction with the SLX4 scaffold protein
Source: DNA Repair (Amst). 2014 Dec;24:131–7. doi: 10.1016/j.dnarep.2014.08.004 (PMC4251979; doi:10.1016/j.dnarep.2014.08.004)
Supplement: Supplementary file 1 [file mmc1.docx]

**Supplementary information Inventory**

**Supplementary Figures**

**Supplementary Figure Legends**

Figure S1: Gateway cloning for generation of yeast two-hybrid constructs

Figure S2: Kanamycin titration to determine optimum concentration for selection of full-length MUS81 fragments

Figure S3: Reverse Y2H to identify MUS81 loss-of-SLX4-interaction mutants

Figure S4: Nuclease assay with 3’ flap substrate

**Extended Experimental Procedures**

Yeast two-hybrid analysis

Generation and selection of point mutations

Complexity of the allele library

Reverse yeast two-hyrbid

Cell culture

Antibodies

DNA constructs

Cell lysis and immunoprecipitation

Virus-based rescue experiments in MEFs and HCT116 cells

Generation of stable HEK293 cell lines

siRNA

Immunofluorescence

**Supplementary figure legends**

**Figure S1: Gateway cloning for generation of yeast two-hybrid constructs**

Steps involved in generating Y2H constructs through the Gateway Cloning system. Full-length or fragments of MUS81 were amplified using primers that contained the attB recombination sites. The PCR products were then recombined into pDONR Express (parent plasmid) to generate pENTR plasmids containing the MUS81 sequence in-frame with a Kan^R^ gene. These were further recombined into the Y2H pDEST32 bait vector, which contains the Gal4-binding domain. Bait plasmids together with the pDEST22-SLX4 prey plasmid were used in the yeast two-hybrid analysis.

**Figure S2: Kanamycin titration to determine optimum concentration for selection of full-length MUS81 fragments**

Kanamycin concentration was determined by transforming TOP10 bacteria with pENTR plasmid encoding MUS81 (1-106) fragment in-frame with the Kan^R^ gene and plating them on medium containing increasing doses of the kanamycin antibiotic. Growth on selection medium containing spectinomycin antibiotic was used as a standard to normalize the number of colonies counted with IPTG induction. All antibiotic concentrations shown were in μg/ml.

**Figure S3: Reverse Y2H to identify MUS81 loss-of-SLX4-interaction mutants**

**(A)** Yeast expressing MUS81 mutants that lacked interaction with SLX4 were selected for by their ability to grow on medium containing 5-FOA (First panel). The yeast were tested for plasmid uptake by plating on medium lacking leucine and tryptophan (Second panel). Loss of interaction was confirmed by plating the yeast on medium lacking leucine, tryptophan and histidine (Third panel). Same as above, except yeast were plated onto X-Gal (Last panel).

**(B)** HEK293 cells were transiently transfected with plasmids expressing FLAG-only, FLAG-MUS81 wild-type (FLAG-MUS81) or FLAG-MUS81 mutants i.e. L25P, L25K, L47P, L47K or L25A L47A. Extracts were subjected to western blotting to test expression (upper panel) or immunoprecipitation with anti-FLAG antibodies (lower panel).

**Figure S4: Nuclease assay with 3’ flap substrate**

Immunoprecipitates from HEK293 cells co-transfected with plasmids expressing FLAG-MUS81 (WT), or nuclease dead mutant FLAG-MUS81 D307A (D307A) and FLAG-EME1 (EME1) were subjected to a nuclease assay with FITC-labeled 3’ flap substrate for time indicated, and the reaction products resolved by gel electrophoresis. FITC-labeled 3’ flap substrate and the cleaved duplex are shown on the right. Western blot confirming the immunoprecipitation with FLAG M2 beads is shown in the left panel.

**Extended Experimental Procedures**

**Yeast two-hybrid analysis**

The Gateway Cloning system (Invitrogen) was used in accordance with manufacturer’s instructions to generate expression vectors, which could be used for testing protein-protein interactions through forward yeast two-hybrid analysis (Fig. S1). Briefly, sequences coding for the full length or a fragment of MUS81 without the stop codon were amplified using primers flanked by the attB recombination sites. The attB flanked PCR products were recombined into the pDONR-Express (Invitrogen) in a reaction mediated by the BP Clonase enzyme (Invitrogen) and selected using the spectinomycin resistance gene. The pDONR vector together with the MUS81 insert is referred to as the pENTR shuttle vector. TOP10 bacteria were electroporated with the BP recombination reaction products and selected using 100 µg/ml of spectinomycin. Transformed bacteria were inoculated in LB + spectinomycin O/N at 37°C and this culture was used to purify pENTR shuttle vector using a QIAprep Spin Miniprep kit. In the pENTR plasmid, MUS81 sequence was recombined in-frame with a kanamycin resistance gene (Kan^R^), which selects against any nonsense or frameshift mutations. Another recombination reaction mediated by the LR recombinase (Invitrogen) is needed to clone the sequence into the Y2H bait vector pDEST32 (Invitrogen) containing the Gal4-binding domain and a LEU2 selectable marker. All constructs were verified by *Eco* RV restriction digest pattern and sequencing. The corresponding yeast two-hybrid pDEST22 construct encoding the full length SLX4 fused to the *GAL4*-activation domain (SLX4-AD) and a *trp1* selectable marker was constructed in a similar manner.

Yeast strain Mav203 (Invitrogen) has a *HIS3* and a *lacZ* gene regulated by Gal4-binding sites. This strain was transformed with a pDEST22 plasmid (Invitrogen) encoding SLX4 fused to the DNA-activation domain of Gal4 (the plasmid also has the TRP1 selectable marker) and with pDEST32 plasmids (Invitrogen) encoding either full-length of fragments of MUS81 fused to the Gal4 activation domain (these plasmids had the LEU2 selectable marker). Empty vectors were used as control. Cells were plated onto minimal medium lacking leucine (LEU) and tryptophan (TRP) and onto synthetic complete medium lacking LEU and TRP and histidine (HIS) to assay reporter gene activation. Cells from the -LEU-TRP plate were overlaid with a nitrocellulose filter that was frozen in liquid nitrogen. The filter was then placed on a piece of filter paper impregnated with X-Gal (5-bromo-4-chloro-3-indolyl-β-D-galactopyranoside) to test for *lacZ* expression that resulted in a blue color.

**Generation and selection of point mutations**

Low fidelity mutagenic PCR was performed to generate mutations using the pDEST32-MUS81 (1-106) as template. As before, the resulting PCR products were recombined into the pDONR-Express vector, which fuses the coding sequence of the MUS81 (1-106) fragment with a kanamycin resistance gene (Kan^R^). The fusion was downstream of an IPTG-inducible promoter. This strategy eliminates mutants with stop codons or frameshift mutations in the MUS81 coding sequence, since these would prevent kanamycin resistance. However, false positive Kan^R^ colonies can appear due to cryptic promoter activity and internal ribosome binding sites. Hence, a threshold concentration of kanamycin needed to be determined to prevent appearance of Kan^R^ colonies independent of an IPTG induction. To this purpose, kanamycin concentration was titrated to yield the highest number of colonies in the presence of IPTG, and minimal or zero background colonies without IPTG. As mentioned earlier, the pDONR plasmid also carries an IPTG-independent spectinomycin resistance gene. The number of colonies counted after spectinomycin selection was taken as the standard yield. An optimal concentration of kanamycin was used to select against truncation mutants (Fig. S2). Frameshift or nonsense mutations that would alter the expression of the fused kanamycin gene were then eliminated through selection on kanamycin plates. In this way, constructs encoding point mutations within an in-frame and complete MUS81 (1-106) fragment were generated.

**Complexity of the allele library**

We wished to ensure that the MUS81 (1-106) allele library was sufficiently complex. To achieve this, conditions for the mutagenic PCR were altered to ensure an approximate mutation frequency of at least 1 mutation per 700 base pairs. As the MUS81 fragment tested was 318 bases long, approximately 0.5 mutations per insert were expected using these conditions. With this frequency of mutation, one would need at least 600 individual clones to cover the entire reading frame in probabilistic terms assuming that no two clones carried the same mutation. However in order to accommodate potential redundancies in the allele library, the complexity of the library needed to be high. Following transformation with the MUS81 allele library, approximately 26000 Kan^R^ colonies were obtained. Given a mutation frequency of approximately 0.5 mutations per insert, this should have resulted in around 13000 clones with a single point mutation in the MUS81 insert. This would reflect complete coverage at least 40 times over. In this way, the entire sequence of the MUS81 (1-106) fragment tested was covered in the mutant allele library.

**Reverse yeast two-hybrid**

The reporter strain Mav203 has, in addition to *HIS3* and *lacZ*, a *URA3* reporter gene driven by *GAL4* sites*.* *URA3* encodes orotidine 5-phosphate decarboxylase (ODCase) that converts 5-fluoroorotic acid (5-FOA) to 5-fluorouracil causing cell death when plated on medium containing 5-FOA and thereby selecting for loss-of-interaction between bait/prey proteins. During reverse yeast two-hybrid assay, cells were additionally plated on -LEU-TRP+5-FOA medium to select for MUS81 mutants that did not interact with SLX4.

**Cell culture**

All cells were kept at 37°C under humidified conditions with 5% CO_2_. HEK293 and 293T cells were grown in DMEM, 10% (v/v) foetal bovine serum FBS, L-glutamine and 1% (v/v) penicilin/streptomycin (10,000 units penicillin and 10 mg streptomycin per ml). For MEFs, medium was supplemented with Na-pyruvate and non-essential amino acids. HCT116 cells were cultured as above in McCoy’s 5a medium supplemented with (FBS) and penicillin/streptomycin stock solution.

**Antibodies**

Antibodies against mouse SLX1 were raised in sheep against full-length mouse SLX1 fused to GST. Antibodies against mouse and human SLX4 were raised against a GST-tagged fragment of relevant SLX4 corresponding to the C-terminal 300 amino acids. Antibodies against mouse MUS81 and EME1 were raised against GST-tagged fragments of the respective mouse proteins corresponding to the first 200 amino acids of each protein. All antibodies were affinity purified using immobilized antigen. Human MUS81 (IQ285) was purchased from Immuquest. Anti-γH2AX S139 (2577L) and anti-GAPDH (14C10) antibodies were purchased from Cell Signalling. All secondary antibodies were purchased from Pierce.

**DNA constructs**

The full-length coding regions for the relevant proteins were generated by PCR using IMAGE consortium EST clones. Point mutations were introduced by quick change mutagenesis. The sequence integrity was confirmed by sequencing analysis.

**Cell lysis and immunoprecipitation**

Cells were lysed in ice–cold buffer: (50 mM Tris-HCl [pH 7.4], 150 mM NaCl, 270 mM sucrose, 1% (v/v) Triton X-100, 1 mM EDTA, 1 mM EGTA, 0.1% ß-mercaptoethanol) with protease inhibitors (Roche) and 50 U/ml of benzonase (Novagen). Lysate was pre-cleared on empty protein G sepharose beads for 30 min at 4°C. All immunoprecipitations were carried out in lysis buffer for 2 h at 4°C. Immunoprecipitations were carried out using 2 µg of primary antibody coupled to 10 µl protein G sepharose per 2 mg of whole cell extract. For FLAG-tagged proteins FLAG-M2 agarose beads (Sigma) were used.

**Virus-based rescue experiments in MEFs and HCT116 cells**

Viruses were produced by co-transfecting 293T cells with the relevant open reading frames cloned into pBABE vector, and with pCMV-VSV-G and pCMV-Gag-Pol expression vectors to direct viral packaging. After 48 hours the virus-containing supernatant was filtered through a 45 µm filter, mixed with polybrene (final concentration of 8 µg/ml) and added to cells (MEFs or HCT116 cells). Three hours later the infection medium was replaced with fresh growth medium. The infection protocol was repeated 24 hours later. Cells were selected for stable expression of relevant proteins with puromycin (3 µg/ml) for 24 hours after the second infection. Protein expression was tested by western blotting.

**Generation of stable HEK293 cell lines**

FlpIn T-Rex HEK293 cells (Invitrogen) stably expressing FLAG-MUS81 (wild-type or mutant) in a tetracycline-inducible manner were made according to the manufacturer’s instructions with relevant MUS81 coding sequence cloned in a pcDNA5-FRT/TO plasmid. Briefly, cells were split into 10 cm dishes and transfected 24 h later using the calcium phosphate method with 1 µg of pcDNA5-FRT/TO plasmid (coding protein of interest) and 9 µg of pOG44 plasmid (Invitrogen). pOG44 expresses the FLP recombinase which allows site specific recombination of the sequence of interest. After 48 h, medium was removed from the cells and replaced with DMEM supplemented with 15 μg/ml blasticidin and 50 μg/ml hygromycin-B. Hygromycin-B was used to select for the integration of the sequence of interest present on the pcDNA FRT/TO plasmid. This media was refreshed every 48 h until colonies began to appear that were visible to eye (approximately 10 days). Drug-resistant colonies were pooled and expanded. Cell lines were tested for expression by the addition of tetracycline at 1 μg/ml 24 h prior to lysis and subsequent western blotting.

**siRNA**

Cells were transfected with the relevant siRNA duplex (10nM) via the calcium phosphate precipitation method. Cells were incubated at 37°C for 48 h. siRNA duplexes were purchased from MWG with a dTdT overhang and the sequences were as follows 5’-3’: Control (UAAUGUAUUGGAACGCAUA), MUS81 #9 (CAGCCCUGGUGGAUCGAUA) and MUS81 #10 (CAUUAAGUGUGGGCGUCUA).

**Immunofluorescence**

HCT116 cells were grown on poly-L-lysine coated 18 mm^2^ square glass coverslips in 6 cm dishes, treated with 20 ng/ml MMC for 16 h and harvested at given time points. Cells were washed twice gently with ice-cold PBS before fixation with ice-cold methanol for 10 min at -20°C and permeabilised with 0.2% Triton X-100 in phosphate–buffered saline for 10 min at room temperature. After several washes in PBS, cells were incubated in blocking solution (PBS containing 3% IgG­–free BSA and 0.2% Tween 20) for 1 h. Coverslips were then incubated with primary antibodies (γH2AX 1:500) in blocking solution at 4°C overnight. After extensive washing in PBS–T (PBS containing 0.2% Tween20), coverslips were incubated with secondary antibodies (Alexa 594 anti-rabbit 1:1000) for 2 h at room temperature and protected from light. Coverslips were washed thoroughly in PBS­–T and cells were stained with DAPI-Hydromount (1:1000) for 10 min at room temperature. Coverslips were then mounted on glass slides and viewed using a deltavision DV3 wide-field deconvolution microscope mounted on a Nikon Diaphot inverted microscope and images were deconvolved after acquisition.
